# Improved Optical Property and Lasing of ZnO Nanowires by Ar Plasma Treatment

**DOI:** 10.1186/s11671-019-3145-1

**Published:** 2019-09-11

**Authors:** Haolin Li, Jilong Tang, Fengyuan Lin, Dengkui Wang, Dan Fang, Xuan Fang, Weizhen Liu, Rui Chen, Zhipeng Wei

**Affiliations:** 1 State Key Laboratory of High Power Semiconductor Laser, School of Science, Changchun University of Science and Technology, 7089 Wei-Xing Road, Changchun, 130022 People’s Republic of China; 2 Department of Electrical and Electronic Engineering, Southern University of Science and Technology, Shenzhen, Guangdong 518055 People’s Republic of China; 3 Centre for Advanced Optoelectronic Functional Materials Research and Key Laboratory of UV-Emitting Materials and Technology, Northeast Normal University, Ministry of Education, Changchun, 130024 People’s Republic of China

**Keywords:** ZnO, Surface-to-Volume Ratio, Laser spectroscopy, Surface modification, Lasing

## Abstract

ZnO nanowires play a very important role in optoelectronic devices due to the wide bandgap and high exciton binding energy. However, for one-dimensional nanowire, due to the large surface to volume ratio, surface traps and surface adsorbed species acts as an alternate pathway for the de-excitation of carriers. Ar plasma treatment is a useful method to enhance the optical property of ZnO nanowires. It is necessary to study the optical properties of ZnO nanowires treated by plasma with different energies. Here, we used laser spectroscopy to investigate the plasma treatments with various energies on ZnO nanowires. Significantly improved emission has been observed for low and moderate Ar plasma treatments, which can be ascribed to the surface cleaning effects and increased neutral donor-bound excitons. It is worth mentioning that about 60-folds enhancements of the emission at room temperature can be achieved under 200 W Ar plasma treatment. When the plasma energy exceeds the threshold, high-ion beam energy will cause irreparable damage to the ZnO nanowires. Thanks to the enhanced optical performance, random lasing is observed under optical pumping at room temperature. And the stability has been improved dramatically. By using this simple method, the optical property and stability of ZnO nanowires can be effectively enhanced. These results will play an important role in the development of low dimensional ZnO-based optoelectronic devices.

## Introduction

As one of the most important semiconductors, zinc oxide (ZnO) is an attractive material for fabrication of optoelectronic devices due to its wide bandgap (3.37 eV) and high exciton binding energy (60 meV) [1, 2]. One-dimensional ZnO nanowires exhibit excellent electrical and optical property which has been widely studied, such as the first optical pumped nanowires laser demonstrated by Yang et. al. [1]. The concept of piezoelectric nanogenerator using ZnO nanowires was first demonstrated by Wang’s research group [3]. Due to the wide range of the conductivity from insulating to highly conducting without external dopants, ZnO nanowire field-effect transistor exhibits excellent performance [4]. When dimension decreases, the quantum confinement effect yields a substantial density of states near the band edges and enhanced radiative recombination due to carrier confinement is achieved. However, for one-dimensional nanostructure like nanowires, due to the large surface to volume ratio, the optical property of the materials is seriously degraded by surface trap states (SS) and surface adsorbed species [5, 6]. Therefore, it is necessary to modify the surface of the low-dimensional materials for improved optical performance.

In order to obtain high-quality ZnO nanowires as light-emitting materials, a lot of surface modifications have been performed, such as coating the nanostructures with different metals [7, 8]. core-shell structure [9–12], polymer covering [13], and plasma-assisted etching. Among them, plasma-assisted etching, due to the convenient operation and cost-effective, is one of the best ways to improve the surface quality of the ZnO nanowires. For plasma-assisted etching, various sources have been employed, such as H_2_ [12, 14–17], Ga^+^ [18], CH_4_ [19, 20], and Ar [21–23]. Among those sources, Ar, as an inert gas, will not induce any chemical reaction to the native material, and therefore it has been chosen to enhance the optical property of ZnO nanowires. Ar plasma treatment is considered to be an effective surface modification technique due to its inexpensive and safety. It is noted that different plasma energies will lead to different surface effects. However, there is no study about the surface treatment by different plasma energies up till now.

In this work, optical property of ZnO nanowires treated by Ar plasma with different energies is investigated. It has been found that the change of the optical properties of ZnO nanowires after plasma treatment can be influenced by various reasons. For low energy plasma treatment, the outer surface cleaning effect plays a dominant role. However, for moderate plasma energy, the further reduction of non-radiative recombination centers and the increased neutral donor-bound exciton (D^0^X) contribute to the improved emission intensity. While for high-plasma energy, reduced optical emission has been observed due to the destruction of material structure. Thanks to the improved optical performance of ZnO nanowires after suitable treatment, optical pumped lasing has been realized at room temperature, and the stability over time of the optical treatment has been proved.

## Methods

### Preparation of ZnO Nanowires

The ZnO nanowires used herein were fabricated using the vapor-liquid-solid technique. ZnO powder and graphite powder with a mass ratio of 1:1 were prepared as the source materials. The mixture was placed in a quartz boat. Au film with a thickness of 3 nm was sputtered on sapphire substrate as catalyst, then transfer on the other quartz boat. In the beginning, the tube furnace was heated to 200 °C with a heating rate of 50 s°C/min. After 15 min, the temperature was raised to 700 °C with a heating rate of 50 °C/min, and then the temperature was kept for 15 min. During the whole progress, Ar gas was introduced for protection, with the gas flow to be 99 mL/min. Then, temperature was raised to 950 °C with a heating rate of 50 °C/min. During this heating progress, O_2_ gas was introduced into the tube furnace with gas flow of 1 mL/min. Keep this condition for 30 min during the growth of ZnO nanowires. Then, decrease the temperature to room temperature under the protection of Ar gas. The sample was then distributed into six parts for subsequent processing.

### Ar Plasma Treatment

For plasma treatment, the Sentech Single Wafer Etching Machine SI 500 ICP with its inductively coupled plasma source (ICP) PTSA200 has been used to etch the ZnO nanowires. In this system, ion density and ion energy can be independently controlled by ICP power and radio frequency (RF) power, respectively. In this work, the ICP power has been set to be 180 W, while the RF power is adjusted from 0 to 400 W to control the energy of the plasma. During the treatment, Ar flux was set to be 25 standard-state cubic centimeters per minute (SCCM) with the pressure of 1 Pa. Processing time for each sample is 90 s. During the whole treatment progress, the temperature of the substrate is maintained at 25 °C.

### Morphology Characterization and Photoluminescence Measurements

The morphology of the nanowire was characterized by Hitachi-4800 field emission scanning electron microscopy (FESEM). Temperature-dependent photoluminescence (PL) measurements were carried out from 50 to 300 K within a closed-cycle helium cryostat. A 325 nm He-Cd gas laser was used as the excitation source. The spot size of the laser beam was about 0.4 cm^2^. The emission was dispersed by Andor SR-500 monochromator, and the signal was detected by an UV-enhanced charged coupled device (CCD). The excitation power of the laser was fixed at 2 mW. For high-density excitation, the signal was collected using the same system, but the excitation source was replaced by a pulsed Nd:YAG fourth harmonic (266 nm) laser with the spot size of the laser beam was about 3 × 10^-4^ cm^2^. The pulse width and the rate of the laser are about 1 ns and 60 Hz, respectively.

## Results and Discussion

**Fig. 1:**
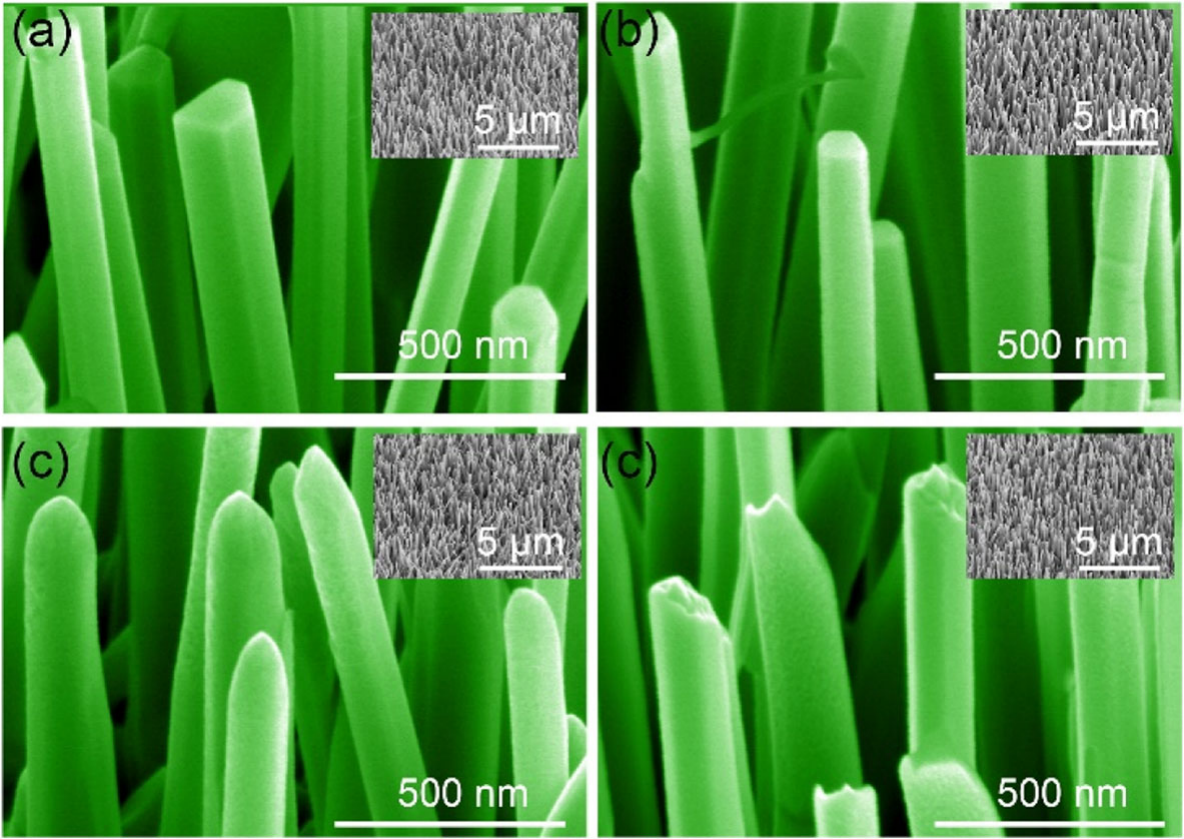
SEM image of the ZnO NWs irradiated by Ar plasma with different energies. **a** As-grown. **b** 0 W. **c** 200 W. **d** 400 W

Structural characterization of the nanowires is shown in Fig. 1. From the SEM image, it can be seen that the diameter of the nanowires is around 170 nm and different plasma energies demonstrate different influences on the surface of nanowires. As shown in Fig. 1a, the as-grown ZnO nanowires are with obvious prismatic structure. With 0 W RF power Ar plasma treatment, the surface of the nanowire has been slightly etched. The nanowires still maintain the prismatic structure, but the outer surface is a little bit rough, which may be ascribed to the high-ion beam energy-induced bombardment. The plasma energy will increase with the increase of the RF power (between 100 and 300 W), and it is noted that the prismatic structure disappeared and is replaced by a circular cross-section as shown in Fig. 1c. When the RF power increases up to 400 W, the plasma energy is large enough to damage the nanowires. This can be confirmed by the breaks of nanowires observed from Fig. 1d. It can be seen from the changes in structural morphology that the changes brought about by different plasma energies on ZnO nanowires can be divided into three processes. With low-plasma energy treatment, a slight surface etch can be used for surface cleaning. When the plasma energy is between 100 and 300 W, it will bring significant morphological changes to the nanowires. This morphological change may affect the optical properties of ZnO nanowires. As the plasma energy increases to 400 W, it will cause irreversible damage to the nanowires.

**Fig. 2:**
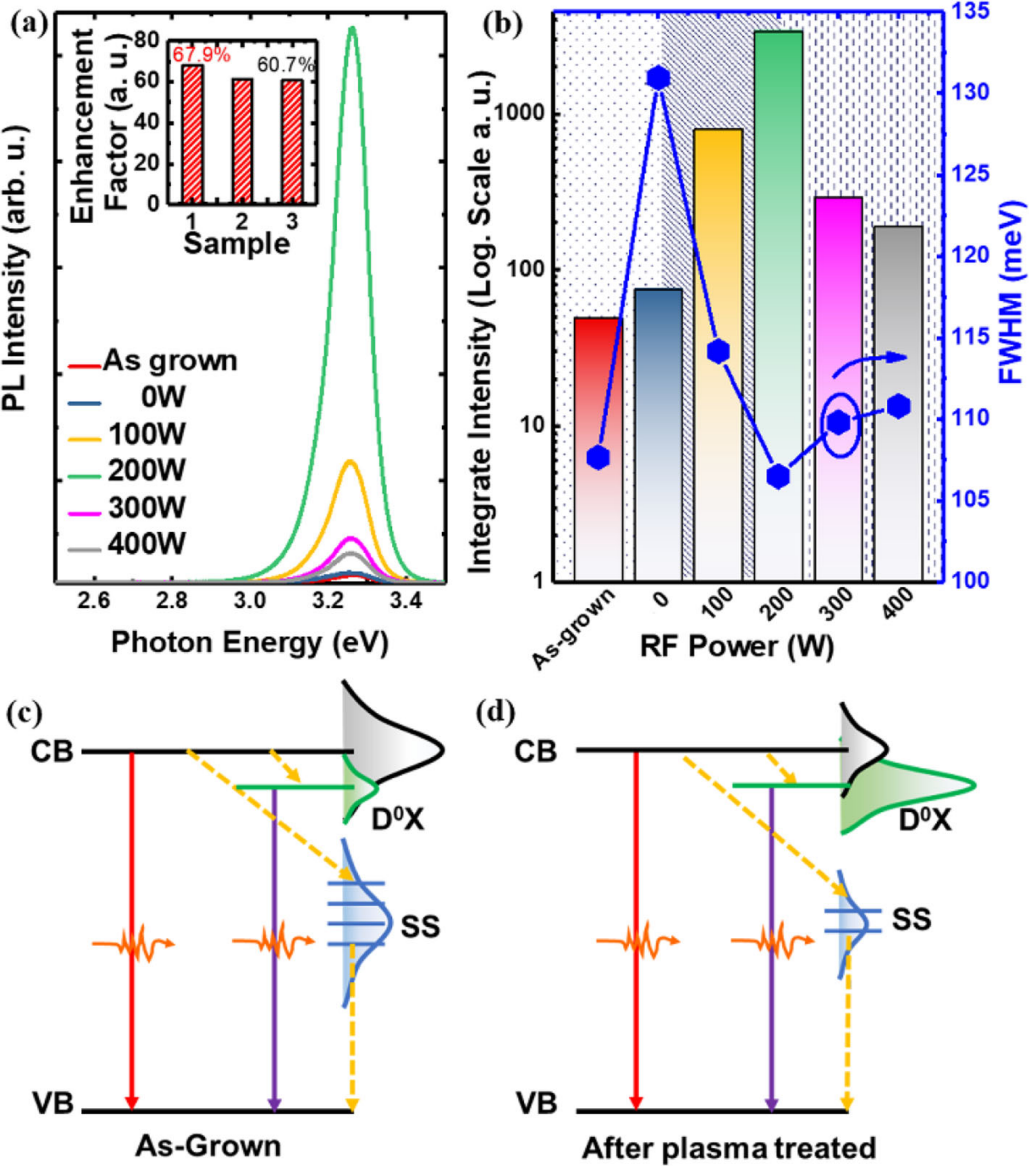
**a** Room temperature PL spectra of the ZnO NWs treated by Ar plasma with different energies (inset shows the repeatability of this treatment). **b** Integrate intensity and FWHM with different energy plasma treated. **c**, **d** Schematic band structure of As-grown sample and after plasma-treated sample

Figure 2 plots the room temperature PL spectrum of the ZnO nanowires before and after Ar plasma treatment. It is noted that the emission intensity of the sample is improved after plasma treatment. When the RF power reaches 200 W, the PL intensity reaches its maximum. Moreover, it is interesting to note that the full width at half maxima (FWHM) also varies with the increase of plasma energy. For example, the FWHM of 0 W treated ZnO nanowires is larger than that of the as-grown one, which can be related to the roughness of the surface observed by SEM. With the increase of plasma energy, the FWHM will decrease until the RF power reaches 200 W. Then, it will increase again when continue to increase plasma energy. Therefore, it is clearly shown that there are different physical mechanisms for the change of optical performance under different treatments, which will be discussed in the following section.

The plasma energy is an important parameter to modify the optical property of ZnO nanowires. For low-energy plasma treatment, surface cleaning effect plays a dominant role. It is well-known that the crystal lattice abruptly terminates at the surface, and the atom in the outermost layer of the surface will have an unpaired electron. The energy levels formed by these unsaturated bonds and other surface-adsorbed impurities together constitute non-radiative recombination centers which appear on the surface of the nanowires and can be removed by the plasma treatment. Plasma cleaning can remove non-radiative recombination centers and deep-level defects located on the outer surface of the nanowires [14, 22, 23], which can explain the increased intensity. The broadened FWHM is due to the roughness introduced by low-energy Ar plasma treatment. For moderate RF power, slight damage has been found, which can be verified from the broadening of the FWHM shown in Fig. 2b. The shallow donor-like defect levels formed by these damages introduce neutral donor levels into the nanowires. The treatment will have a positive effect on the emission due to the further reduction of non-radiative recombination centers and the increased neutral donor levels. For high power, the plasma treatment will induce damage to the nanowire, which will be responsible for the decrease of intensity and the increase of FWHM. From the investigation carried out herein, it is found that the most suitable condition is the RF power treatment at 200 W. More importantly, the same experimental condition is used for three different samples, and it is found that both of them show similar 60-folds enhancements of the emission at room temperature, which confirms the high repeatability of the treatment.

**Fig. 3:**
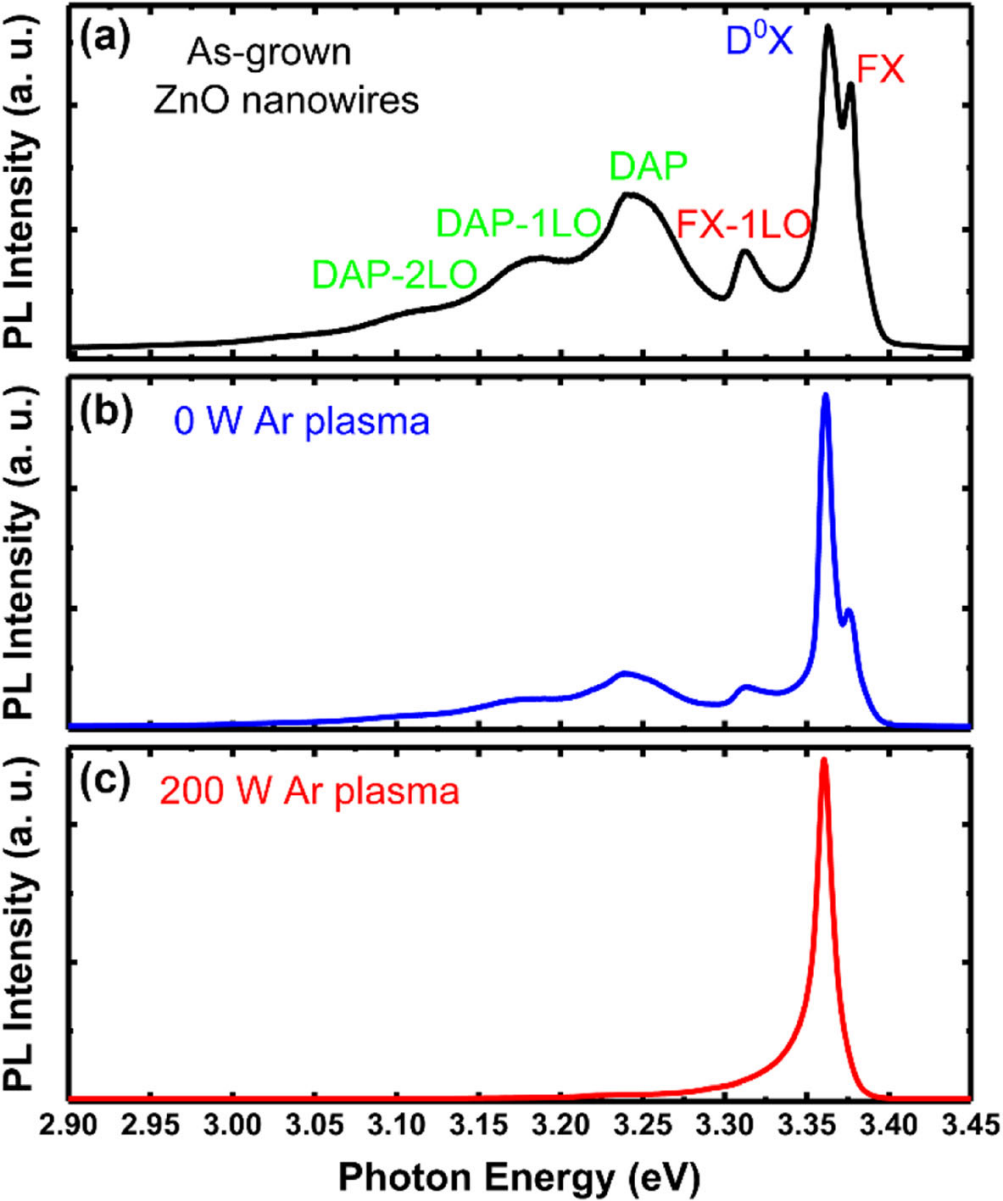
Low-temperature PL spectra of the ZnO NWs treated by Ar plasma with different energies. **a** As-grown. **b** 0 W. **c** 200 W

To further confirm the origin of the emission of ZnO nanowires treated by different energy plasma, PL measurement at low temperature (50 K) was carried out. As it is shown in Fig. 3a, the dominated emission of the sample comes from D^0^X located at 3.363 eV [24, 25]. At the higher energy region, the peak at 3.377 eV can be ascribed to the free exciton (FX) emission and its longitudinal optical (LO) phonon replica can also be clearly identified. At the lower energy region, peaks localized at 3.241 eV, 3.171 eV, and 3.101 eV can be ascribed to the recombination of donor-acceptor pair (DAP) and its LO phonon replicas. In Figure 3b, the peak position of ZnO nanowire treated by 0 W Ar plasma shows similar emission with the as-grown sample. A weaker DAP emission has been observed, which implies the removal of donor or acceptor impurities on the surface of ZnO. Then, with RF power reaches 200 W, DAP emission disappears. As it can be seen from Figure 3c, the modified ZnO nanowires only show an emission located at 3.361 eV with no apparent FX emission and DAP emission. The asymmetrical shape of the emission is due to the existence of phonon replicas, which indicates that all electrons are captured by the neutral donor level. A similar observation was also reported for ZnO treated with H plasma, and they attributed the peak to H doping. However, in this work, no H plasma was introduced during the experiment. Considering the peak is close to the D^0^X peak position of the untreated sample at low temperatures (only 2 meV between them), we believe that this peak also comes from D^0^X, which can be confirmed by the temperature-dependent peak position discussed next. With the Ar plasma treatment reaches 200 W, the DAP peak disappears, while enhanced D^0^X emission has been observed. Therefore, it can be concluded that plasma treatment can remove the acceptor impurities and introduce more donor-bound excitons. Meanwhile, the removal of some non-radiative recombination centers on the surface also counts for the enhanced emission.

**Fig. 4:**
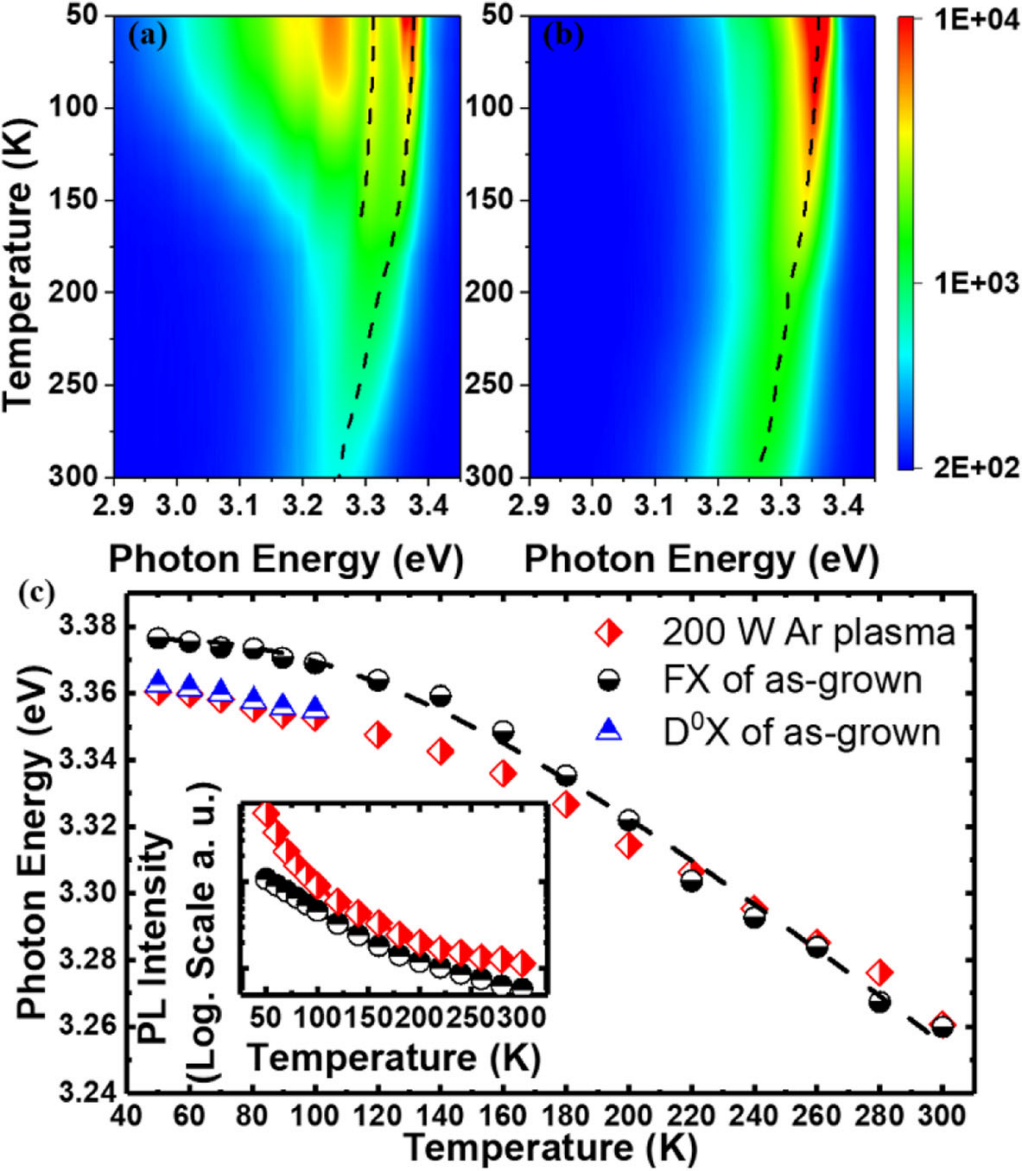
**a**, **b** Temperature-dependent PL spectra of the As-grown ZnO NWs and irradiated by 200 W Ar plasma. **c** Photon energy and PL emission from the as-grown sample

To better understand the origin of the emission from ZnO nanowires, temperature-dependent PL measurement has been investigated. For as-grown ZnO nanowires, with temperature increase the intensity of D^0^Xdecrease quickly and totally disappear at temperature ~ 100 K, and FX exists for the whole temperature range (50–100 K). It also can be seen that the DAP emission and its LO phonon replicas show a slight blue shift with temperature, which is the characteristic for DAP [24]. As for 200 W Ar plasma treated ZnO nanowires, only one peak exists during the whole temperature range, and this peak shows a red shift with temperature. To better understand the temperature-dependent optical properties of the sample, emission peak position and intensity of the ZnO nanowires before and after 200 W Ar plasma treatment were presented in Fig. 4b. As is shown, the photon energy of the FX can be well-fitted with Bose-Einstein relation [26–28].

1 $$ E(T)=E(0)-\frac{\lambda }{\exp \left(\frac{\mathrm{\hslash}\omega }{k_BT}\right)-1} $$

where *E(0)* is the bandgap at 0 K, *λ* is the proportional coefficient, *ℏω* is the effective phonon energy, and *k*_*B*_ is the Boltzmann constant. For the as-grown sample, *E(0) =* 3.376 eV, *λ* = 359 meV, *ℏω* = 35 meV. The effective phonon energy of this sample is in good agreement with the energy maximum of the low-energy group of bulk phonon density of states (8 THz = 33 meV or 380 K) [28].

The emitted photon energy from the 200 W Ar plasma-treated sample shows a different trend with temperature. It follows D^0^X at low temperature, while the temperature reaches about 180 K, the peak position shows a similar trend with FX. The conversion point is intensity change with temperature close to the binding energy of D^0^X (*E*_*b*_ *= E(FX)−E(D*^*0*^*X*) = 16 meV or 185 K). And as shown in the inset of Fig. 4c, the emission intensity of the 200 W Ar plasma-treated sample decreases sharply at low temperature which is consistent with the characteristics of D^0^X. Based on the discussion above, for moderate plasm energy, more neutral donor levels have been introduced into the nanowires, which dominate the emission at low temperature. The passivation of surface dangling bonds and non-radiative recombination centers on the surface also counts for the enhanced emission.

**Fig. 5:**
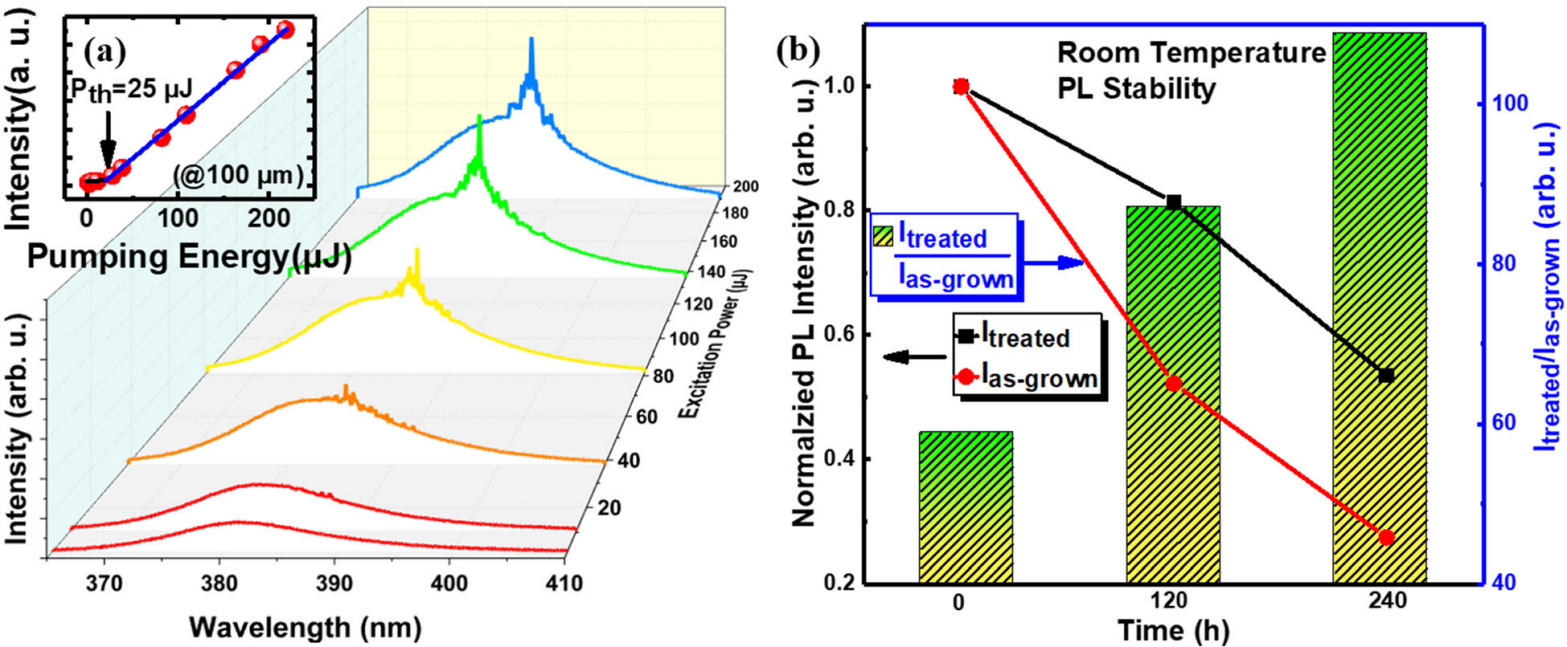
**a** Lasing under optical pumping from ZnO NWs irradiated by 200 W Ar plasma. **b** Stability of ZnO NWs (the intensity ratio after plasma treatment over time compared with the as-grown samples)

The sample under high-density optical pumping by a pulse laser at room-temperature was performed and the data are shown in Fig. 5. The lasing phenomenon was not observed in the as-grown sample. However, in the 200 W Ar plasma-treated sample, when the energy exceeds the threshold ~ 25 μJ, sharp peaks emerge from the low-energy shoulder of the broad spontaneous emission. The lasing emission at 390 nm can be ascribed to the P-band emission of ZnO [29] or significant self-absorption effect [30]. The integrated PL intensity of these stimulated peaks with respect to pump density is shown in the inset of Fig. 5a. The non-linear increased intensity is a characteristic of lasing [1, 31]. The lasing phenomenon of plasma-treated samples is also based on the reasons mentioned above, after plasma treatment, surface trap states can be removed, and the optical loss has been minimized to achieve population inversion after optical pumping. What is more, thanks to the passivation of Ar plasma, the ZnO nanowires treated by 200 W Ar plasma exhibit better stability than the as-grown one. As can be seen from Fig. 5b, it can be seen that the ratio after plasma treatment gradually increases over time compared with the as-grown samples. This implies that the sample after plasma treatment has higher stability.

## Conclusions

In summary, the optical property of ZnO nanowires treated by Ar plasma with different energies is investigated. We have found that the enhancement of the optical properties of ZnO nanowires after plasma treatment is the result of various reasons. The best processing condition is 200 W RF power. For low-energy plasma treatment, outer surface cleaning effect plays a dominant role, which leads to the increased intensity and the broadened FWHM. Under moderate RF power, the treatment will have a positive effect on the PL due to the further reduction of non-radiative recombination centers and the increased neutral donor levels. The neutral donor level can capture carriers and enhance optical emission. When the plasma energy exceeds the threshold, it will bring irreparable damage to the ZnO nanowires. Due to the improvement of optical properties of ZnO nanowires, optically pumped lasing has been realized from the suitably treated ZnO nanowires at room temperature, and the stability over time of the optical treatment has been proved. By investigating the effect of plasma energy on the optical property of ZnO nanowires, we have found a simple and effective way to improve the optical property of ZnO nanowires, which will inject new vitality for the development of extreme ultraviolet optoelectronic devices.

## Data Availability

The authors declare that materials and data are promptly available to readers without undue qualifications in material transfer agreements. All data generated in this study are included in this article.

## Abbreviations

CCD: Charged coupled device
D^0^X: Neutral donor-bound exciton
DAP: Donor-acceptor pair
FESEM: Field emission scanning electron microscopy
FWHM: Full-width at half maxima
FX: Free exciton
ICP: Inductively coupled plasma
LO: Longitudinal optical
PL: Photoluminescence
RF: Radio frequency
SCCM: Standard-state cubic centimeters per minute
SS: Surface trap states
